# Common themes and unique proteins for the uptake and trafficking of nickel, a metal essential for the virulence of *Helicobacter pylori*

**DOI:** 10.3389/fcimb.2013.00094

**Published:** 2013-12-09

**Authors:** Hilde de Reuse, Daniel Vinella, Christine Cavazza

**Affiliations:** ^1^Unité Pathogenèse de Helicobacter, Département de Microbiologie, Institut Pasteur, ERL CNRS 3526Paris, France; ^2^Metalloproteins Group, Institut de Biologie Structurale Jean-Pierre Ebel, UMR 5075, CEA, CNRS, Université Joseph Fourier-Grenoble 1Grenoble, France

**Keywords:** nickel, urease maturation, hydrogenase, *Helicobacter pylori*, metal trafficking

## Abstract

Nickel is a virulence determinant for the human gastric pathogen *Helicobacter pylori*. Indeed, *H. pylori* possesses two nickel-enzymes that are essential for *in vivo* colonization, [NiFe] hydrogenase and urease, an abundant virulence factor that contains 24 nickel ions per active complex. Because of these two enzymes, survival of *H. pylori* relies on an important supply of nickel, implying a tight control of its distribution and storage. In this review, we will present the pathways of activation of the nickel enzymes as well as original mechanisms found in *H. pylori* for the uptake, trafficking and distribution of nickel between the two enzymes. These include (i) an outer-membrane nickel uptake system, the FrpB4 TonB-dependent transporter, (ii) overlapping protein complexes and interaction networks involved in nickel trafficking and distribution between urease and hydrogenase and, (iii) *Helicobacter* specific nickel-binding proteins that are involved in nickel storage and can play the role of metallo-chaperones. Finally, we will discuss the implication of the nickel trafficking partners in virulence and propose them as novel therapeutic targets for treatments against *H. pylori* infection.

## Introduction

For many organisms, acquisition of essential metal ions that are present at low concentrations in their environment is a critical process. Once acquired, the correct metal has to be delivered and incorporated into specific target enzymes through dedicated protein complexes comprising chaperones and so-called accessory proteins and when in excess the metals have to be stored as their non-physiological high intracellular concentrations are toxic.

The proteins involved in import, cellular storage, distribution, and incorporation of metal ions into enzymes are collectively referred to as “metal trafficking proteins”. In bacteria, tremendous efforts have been devoted to decipher the mechanisms of iron uptake, trafficking and intracellular homeostasis control. Much less information is available for the acquisition and homeostasis of nickel, which is nevertheless an essential element for several bacteria. Nickel is the cofactor of at least nine enzymes involved in diverse cellular processes such as energy metabolism or virulence, including [NiFe]-hydrogenase, urease, Ni-SOD, CO-dehydrogenase (Higgins et al., 2012; Boer et al., 2013). Nickel is ubiquitously found in the environment and present at low concentrations in vertebrates. Although no enzymes or co-factors that include nickel were identified in higher organisms so far, animal experiments with nickel-deficient diets suggest a physiological role for nickel in these organisms (Denkhaus and Salnikow, 2002). It is tempting to speculate that nickel is required in higher organisms to sustain growth of the microbiote. Finally, different mechanisms have been proposed to explain cellular nickel toxicity, among which replacement by nickel of the metal of essential metalloproteins or indirect generation of oxidative stress (Macomber and Hausinger, 2011).

**Figure 1:**
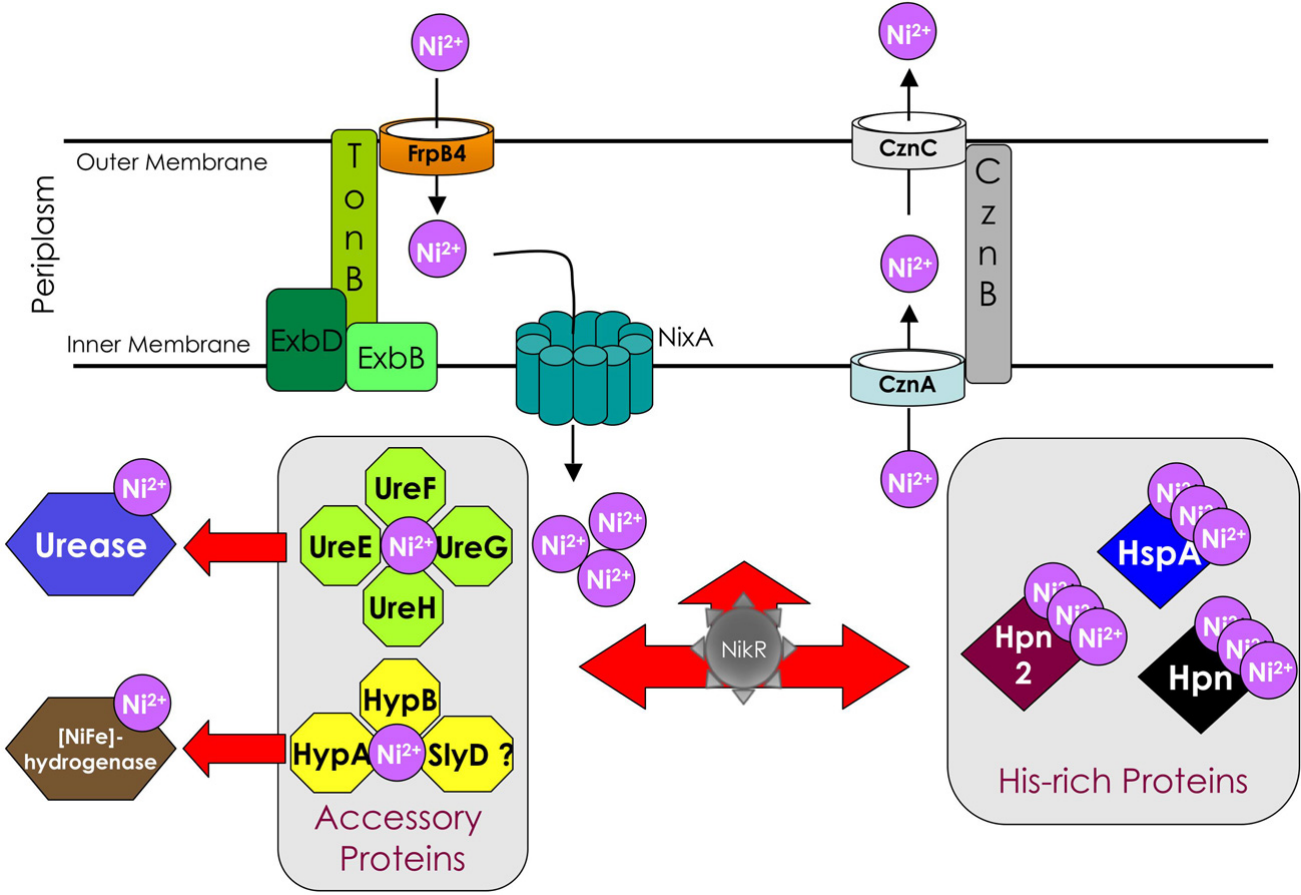
**Schematic representation of the *Helicobacter pylori* major components involved in nickel uptake, efflux, trafficking and incorporation into the two nickel metalloenzymes urease and [NiFe] hydrogenase and of the nickel-responsive transcriptional regulator NikR**.

As for iron, specific and controlled nickel transport, efflux and trafficking processes are necessary as well as specific nickel storage proteins and nickel-responsive transcriptional regulators (such as NikR, for a review see Dosanjh et al., 2009). The gastric pathogen *Helicobacter pylori* presents interesting nickel management strategies, some being common to other bacteria and others being unique to this organism (Figure 1). *H. pylori* is therefore a fascinating model organism to study nickel uptake and trafficking as well as the link between virulence and these pathways. These aspects will be discussed in this mini-review.

## Nickel, a virulence determinant for *helicobacter pylori*

*H. pylori* is a gram-negative bacterial pathogen of major importance that colonizes the stomach of about half of the human population worldwide. Gastric infection by *H. pylori* causes gastritis and peptic ulcer disease. In addition, decades of persistent infection by *H. pylori* favors the development of gastric cancer (Wroblewski et al., 2010). *H. pylori*, is until now the sole bacterium recognized as a class I carcinogen and causes 800,000 deaths worldwide annually. It is the only bacterium that can multiply in the stomach, a hostile acid niche (median pH 2). Nickel can be regarded as a virulence determinant for *H. pylori* since it is the co-factor of urease, an enzyme essential for resistance to acidity. Urease is a major virulence factor essential for colonization of animal models. [NiFe] hydrogenase, the only other nickel-enzyme found in *H. pylori* is important for murine colonization probably because it enables the use of H_2_ as an energy source. Urease represents 6–10 % of total *H. pylori* proteins. In addition, we measured a total intracellular nickel concentration of approximately 60 nM in *H. pylori*, corresponding to 50 times that of *Escherichia coli* (Schauer, 2007). Thus, a constant and important supply of nickel is required for the survival of *H. pylori* within the stomach, implying a tight control of its acquisition, distribution and storage. The properties of urease and hydrogenase will be briefly presented followed by the description of the nickel trafficking pathways.

## Urease and hydrogenase

### Urease

Urease catalyzes the hydrolysis of urea to produce ammonia and carbamate, the latter being spontaneously degraded into a second molecule of ammonia and bicarbonate (Boer et al., 2013). These compounds act as buffers to maintain a neutral cytosolic pH in *H. pylori* (Stingl and De Reuse, 2005). The first crystal structure of a bacterial urease, from *Klebsiella aerogenes*, revealed a dinuclear nickel active site deeply buried in the alpha subunits (Jabri et al., 1995). The two nickel ions are bound to the protein *via* two histidines (His), an aspartate and a bridging carboxylate group of a carbamylated lysine. The *H. pylori* urease is composed of the UreA and UreB subunits. Its structure has also been solved (Ha et al., 2001) revealing a giant 1.1-MDa dodecameric complex (four trimers of UreA-B heterodimers) containing as much as 24 nickel ions. This enzyme has the highest affinity to its substrate ever described for a urease.

### Hydrogenase

[NiFe]-hydrogenases catalyze the reversible heterolytic cleavage of dihydrogen. The active site, buried in the large subunit of a heterodimeric protein, contains a Fe(CO)(CN)_2_ unit and a nickel ion (Fontecilla-Camps et al., 2007). Protein coordination is mediated by two Fe-Ni bridging and two nickel-binding terminal Cys thiolates (Volbeda et al., 1995). The catalytic nickel ion has both a terminal coordination site and a bridging coordination site. *H. pylori* possesses a membrane-bound respiratory [NiFe] hydrogenase (Maier et al., 1996) that catalyzes the oxidation of dihydrogen. It is important for colonization of the mouse model possibly because dihydrogen, a fermentation by-product of the gut microorganisms, is used by *H. pylori* as an energy substrate (Olson and Maier, 2002; Maier, 2005).

## *in vivo* urease activation and accessory protein complexes

*In vivo* activation of urease requires dedicated accessory proteins that function through protein complexes to help the specific incorporation of nickel at the right site in a coordinated manner (Carter et al., 2009). In *H. pylori*, the *ureA*-*ureB* structural genes are followed by the accessory gene cluster *ureEFGH*. Using Tandem Affinity Purification, we isolated from *H. pylori* cells a complex comprising UreA-B and the complete activation complex UreH-UreF-UreG-UreE (Stingl et al., 2008). A computational model of this complex was recently proposed (Biagi et al., 2013).

UreE is a nickel metallochaperone that interacts with UreG (Bellucci et al., 2009). UreG is an intrinsically unstructured GTPase that dimerizes upon binding of a metal ion (Zambelli et al., 2005, 2009; Musiani et al., 2013). It has been suggested that binding of the UreF-UreH complex induces conformational changes in urease, allowing nickel ion and carbon dioxide to access the active site. UreF was shown to gate the GTPase activity of UreG to enhance the fidelity of urease metallocenter assembly (Boer and Hausinger, 2012). A recent crystal structure of the *H. pylori* UreG-UreF-UreH complex reveals how UreF and UreH facilitate UreG dimerization and how it assembles its metal binding site (Fong et al., 2013). The addition of nickel and GTP to the UreG-UreF-UreH complex causes release of the UreG dimer that binds nickel at the dimeric interface. *In vitro*, nickel-charged UreG dimer was shown to activate urease in the presence of UreF/UreH. How nickel is transferred from UreE to the binding site of UreG and how the complete activation complex interacts with urease has still to be determined.

### *in vivo* hydrogenase activation and interconnectivity between the urease and hydrogenase maturation pathways in *H. pylori*

[NiFe] hydrogenase activation by nickel incorporation also requires dedicated accessory proteins that are conserved in *H. pylori* (Leach and Zamble, 2007; Maier et al., 2007). HypCDEF are necessary for the synthesis and correct insertion of the Fe(CO)(CN)_2_ moiety in apo-hydrogenase. Once the iron center gets assembled and inserted, nickel is delivered to the enzyme through the concerted action of the metallo-chaperone HypA and the GTPase HypB that were characterized in *H. pylori* (Xia et al., 2012). In *E. coli*, the nickel-binding protein SlyD is an essential additional partner of hydrogenase maturation, presumably involved in stimulating the release of nickel from HypB (Leach et al., 2007).

A unique particularity of nickel trafficking in *H. pylori* is the interconnectivity between the urease and hydrogenase maturation pathways. Indeed, *H. pylori* mutant characterization revealed that HypA and HypB are required not only for hydrogenase maturation but also for full urease activation (Olson et al., 2001). In agreement with this, our *H. pylori* interactome analysis evidenced that HypB is physically associated to the urease maturation complex (Stingl et al., 2008). This interactome also contained SlyD (Stingl et al., 2008) shown to interact with HypB *in vitro* and *in vivo* in *E. coli* (Cheng et al., 2013). However, the role of SlyD in *H. pylori* cells and in urease/hydrogenase activities remains unclear. Benanti and Chivers (2009) showed that in *H. pylori* strain 26695, urease activity was strongly diminished in a double Δ*slyD*/Δ*hypA* mutant as compared to a single Δ*hypA* mutant, but this effect was not observed in strain G27. While SlyD may serve as a nickel reservoir to activate urease, the properties of nickel storage proteins might differ depending on the *H. pylori* genetic context. To be mentioned, in the closely related organism *Campylobacter jejuni*, hydrogenase activity is not modified in a Δ*slyD* mutant (Howlett et al., 2012).

Using optical-biosensing methods, it was found that the *H. pylori* HypA and UreG proteins compete with each other for UreE recognition, suggesting that the function of HypA in urease activation relies on nickel delivery or exchange rather than on catalytic activities (Benoit et al., 2012). Indeed, purified recombinant HypA is sufficient for the recovery of urease activity in *H. pylori* cell lysates from a *hypA* deletion mutant (Herbst et al., 2010). This can be related to the observation that unlike many of its orthologs, the *H. pylori* UreE dimer only binds one equivalent of Ni^2+^ (Bellucci et al., 2009).

While the accessory proteins for both urease and hydrogenase maturation are conserved in *H. pylori*, several particularities of nickel trafficking in this organism have emerged. At least three nickel-binding proteins have important functions in nickel trafficking in *H. pylori* and a novel nickel uptake system was discovered. We believe that *H. pylori* acquired these additional partners to deal with its exceptionally high intracellular nickel concentration, the fluctuating nickel availability and pH in the stomach and with a critical need to coordinate nickel distribution between two essential enzymes.

### Nickel transport and efflux

In Gram-negative bacteria, energized transport of metabolites such as iron-siderophore complexes through the outer membrane (OM) relies on the TonB machinery and on TonB-dependent-transporters (TBDTs). The first nickel transport system across a bacterial OM was described in *H. pylori* by our group (Schauer et al., 2007). Its expression is repressed by NikR in the presence of nickel. This transport requires the FrpB4 TBDT and is acid-induced, allowing *H. pylori* to optimize urease activity by nickel incorporation under conditions where urease activity needs to be maximal. Additional mechanisms certainly exist allowing, for example, nickel entry at neutral pH. FecA3, another *H. pylori* TBDT whose synthesis is under nickel control (Romagnoli et al., 2011), is probably an alternative nickel-uptake system (Ernst et al., 2006). It is not clear under which form nickel is recognized by the TBDT. By analogy with siderophores, it is possible that a nickelophore, *i.e*., a small organic chelator of nickel, is required for its transport. A nickelophore was shown to be required for nickel binding into the *E. coli* NikA protein: it can be either a small organic ligand (Cherrier et al., 2008) or a (L-His)_2_ (Chivers et al., 2012; Lebrette et al., 2013).

In *H. pylori*, nickel transport across the cytoplasmic membrane can be mediated by NixA, a high-affinity and low-capacity nickel transporter (Fulkerson and Mobley, 2000) of the NiCoT family, which expression is repressed by nickel (Wolfram et al., 2006). As a NixA mutant retains half of urease activity and is still able to colonize the mouse model (Nolan et al., 2002), alternative ways of nickel entry must exist. Other *Helicobacter* species present different combinations of similar nickel transport systems. In *Helicobacter mustelae*, nickel uptake and urease activation depend on NikH, a TBDT different from FrpB4 (Stoof et al., 2010b) and on the FecDE/CeuE ABC transport system (Stoof et al., 2010a). *Helicobacter hepaticus* possesses genes regulated by NikR that are homologous to those encoding the *E. coli* nickel ABC transporter and to *nikH* of *H. mustelae* (Benoit et al., 2013b). The *fecDE/ceuE* genes are conserved in *H. pylori* and might be an additional nickel transport system across the inner membrane.

Again, the possible variety in nickel uptake mechanisms underscores the utmost importance of nickel uptake in *H. pylori* and closely related *Helicobacter* species.

Having crossed the inner membrane, nickel has to be directed to its proper targets while avoiding potential damages caused by free metal ions. If nickel is in excess with respect to *H. pylori* cellular needs, it must either be stored or exported from the cell. Only one nickel export system has been described in *H. pylori*, a proton-driven RND-type metal efflux-pump encoded by the *cznABC* genes. Inactivation of this pump increases *H. pylori* sensitivity to nickel, cadmium and zinc and impairs colonization of the gerbil stomach (Stahler et al., 2006), underlining the importance of metal homeostasis for *H. pylori* virulence.

### Unusual nickel chaperones and storage proteins in *H. pylori*

In *H. pylori*, HspA is the sole member of the highly conserved and essential GroES co-chaperonine family (Suerbaum et al., 1994). The *hspA* gene transcription is activated by NikR with nickel (Muller et al., 2011). HspA protein is particular by the fact that it contains a His- and Cys-rich C-terminal extension that was shown to bind nickel ions *in vitro* (Kansau et al., 1996). Deletion of this extension is viable and impairs the maturation of hydrogenase but not that of urease (Schauer et al., 2010). We concluded that HspA could constitute a nickel storage pool specifically used for hydrogenase maturation. How nickel is mobilized from HspA and whether HspA provides nickel to other proteins remain to be determined. A more general role of HspA in nickel storage/detoxification is suggested by its abundance and by the fact that deletion of its C-terminal extension decreases the intracellular nickel content and increases nickel sensitivity (Schauer et al., 2010).

*H. pylori* also possesses two proteins of remarkable amino-acid composition that are conserved in *H. pylori* and have no orthologs outside the *Helicobacter* species. Hpn and Hpn-2 (or Hpn-like) are two small proteins (7 and 8 kDa) that are extremely rich in His-residues: 47 and 25 % of the total residues, respectively. Hpn-2 contains additional poly-Glutamine stretches representing 40% of the total residues. These two proteins were shown *in vitro* to bind nickel and to form multimers (Gilbert et al., 1995; Ge et al., 2006; Rowinska-Zyrek et al., 2011; Zeng et al., 2008, 2011). Like *hspA*, transcription of these genes is upregulated by NikR with nickel (Muller et al., 2011). Because *hpn* and *hpn-2* mutants were found to be more sensitive to high exogenous nickel concentrations than a wild type strain, these abundant proteins were suggested to be involved in nickel storage and detoxification *via* sequestration of excess nickel (Mobley et al., 1999; Seshadri et al., 2007). Seshadri et al. (2007) reported that both proteins compete with the nickel-dependent urease maturation machinery under low nickel conditions. However, previous data showed wild-type urease activity in a *hpn* deletion mutant (Gilbert et al., 1995). While Hpn and Hpn-2 proteins are certainly central in the nickel trafficking pathways of *H. pylori* and are possibly directly or indirectly involved in urease activation, their respective roles in these processes remain to be established. Recently, purified Hpn was shown to form *in vitro* amyloïd-like fibrils that are toxic when applied to cultured gastric epithelial cells (Ge et al., 2011). The existence and function of these fibers and the effect of nickel on their formation has yet to be demonstrated *in vivo*.

### Urease, nickel, and virulence

The two nickel metalloenzymes (urease and [NiFe] hydrogenase) are determinant in *H. pylori* colonization capacity. In addition to its role in acid resistance, urease fuels *H. pylori* with ammonium for nitrogen assimilation (Williams et al., 1996). The produced ammonia is cytotoxic either alone or in conjunction with neutrophil metabolites (Sommi et al., 1996). Urease activity is also important for survival into macrophages, evasion from phagocytosis, and complement-mediated opsonisation. Purified urease protein stimulates activation of macrophages, dysregulates tight-junctions and induces cytokine production from gastric epithelial cells. These effects might be related to the large amounts of urease-bound nickel delivered to the host cells, considering the 24 nickel ions per active urease complex.

*H. pylori* displays a chemotactic repulsive response to nickel that might help its orientation during stomach colonization (Sanders et al., 2013). In addition, using a mouse model fed with Ni-deficient chow, a weak colonization defect was observed with the double Δ*hpn-Δhpn-2* mutant (Benoit et al., 2013a). This suggests a role of Hpn and/or Hpn-2 in nickel incorporation during host colonization. Incubation of gastric epithelial cells with purified recombinant *H. pylori* SlyD protein disturbs cell proliferation, apoptosis, and enhances cell transformation and invasion (Kang et al., 2013). It was thus proposed that SlyD contributes to the gastric pathogenicity of *H. pylori*. Further studies with *slyD*-deficient strains are needed to establish the role of SlyD *in vivo*. Finally, purified recombinant HspA protein induces the expression of pro-inflammatory cytokines in human cells (Lin et al., 2006). Here again, nickel delivery to hosts cells mediated by nickel-binding proteins might cause these effects.

## Conclusion

*H. pylori* possesses original properties for nickel uptake and trafficking that are directly related to its virulence and reflect the absolute need for this organisms to manage high nickel concentrations. Some of the factors that were discovered in *H. pylori* seem to be present in other organisms (nickel TBDTs in other *Helicobacter* species) and such transporters are predicted in other species (Schauer et al., 2008).

Several fascinating questions remain to be answered. Are the functions of Hpn and Hpn-2 proteins restricted to nickel storage or do they constitute urease-dedicated *H. pylori* chaperones? How large is the variety of nickel transporters? Are there nickelophores needed for nickel uptake and, if so, is this nickelophore synthesized by *H. pylori* or acquired from the environment? Given the properties of *H. pylori* and the absence of nickel-enzymes in the human body, it is tempting to propose the nickel trafficking pathways of *H. pylori* as targets for the development of alternative antibacterial drugs.

### Conflict of interest statement

The authors declare that the research was conducted in the absence of any commercial or financial relationships that could be construed as a potential conflict of interest.
